# Seasonal Influenza: Waiting for the Next Pandemic

**DOI:** 10.4103/0974-777X.52983

**Published:** 2009

**Authors:** Angela Clem, Sagar Galwankar

**Affiliations:** *University of South Florida, Department of Global Health, Tampa, Florida, USA*

**Keywords:** Influenza, Pandemic, Seasonal

## Abstract

With the ongoing cases of H1N1 influenza (aka Swine Flu) occurring around the globe, seasonal influenza has a tendency to be overlooked by the media and general population as a source of illness and death. Yet, these pandemic influenza viruses arise from these seasonal influenza viruses. This article will provide an overview of seasonal influenza, its prevention and treatment, and the global surveillance system in place, used to detect the next influenza pandemic.

## INTRODUCTION

With the ongoing cases of H1N1 influenza (aka Swine Flu) around the globe, seasonal influenza has a tendency to be overlooked. However, seasonal influenza contributes significantly to global morbidity and morality and is the source of these influenza pandemics. This article will provide an overview of seasonal influenza, its prevention and treatment, and the global surveillance system used to detect the next influenza pandemic.

## BASIC OVERVIEW OF INFLUENZA

There are three types of influenza viruses - A, B, and C. However, only Influenza A and B viruses are known to cause an epidemic in humans.

Type A virus is further sub-divided, based on the typing of two important surface glycoproteins, hemagglutinin (HA) and neuraminidase (NA). There are 16 known HA sub-types and nine known NA sub-types of influenza A virus, which can recombine to create novel combinations of influenza. In addition, there are eight separate gene segments composing influenza A virus. The influenza A sub-type and the influenza B virus are further classified into strains.[1]

Re-assortment of these gene segments allows the development of novel Influenza A virus that can cause a pandemic due to the lack of immunity. This major antigenic change is called antigenic shift. In contrast, seasonal influenza viruses exhibit frequent point mutations which lead to more gradual shifts in their genomes. This is known as antigenic drift; and, it is the reason that new influenza vaccines must be prepared yearly. Antibodies produced against the previous strain may not protect against the altered virus.

Influenza A virus can infect numerous species including pigs, whales, horses, seals, and humans. However, these viruses are typically species-specific and do not normally cross the species barrier. As an example, H1N1 and H3N2 sub-types are known to cause outbreaks in pigs. As noted with the ongoing H1N1 epidemic (Swine flu) and avian influenza, these viruses can cross the species barrier.[1]

Wild birds are the exception to the specificity of influenza A virus. Wild birds are the natural reservoirs for all subtypes of influenza A virus; and, these birds are typically unaffected by the viruses which allows them to be frequent sources of Influenza transmission.

In contrast to Influenza A, humans are the only known reservoirs of Influenza B virus. Influenza B strains have caused epidemics in the past but never pandemics. The Influenza B viruses that are currently circulating have been classified into two distinct genetic lineages known as Yamagata and Victoria. These viruses undergo antigenic drift less frequently than Influenza A. 2 As for Influenza C, it is known only to cause mild illness in humans.[1]

Influenza virus can be quite resistant to environmental factors. For example, the virus can survive in contaminated manure for at least three months, in cool climates; and, one gram of this infected manure can contain enough viral particles to infect one million birds. In addition, the virus can also survive in water for up to four days at 72°F, greater than one month at 32°F, and potentially indefinitely in frozen material.[1]

## EPIDEMIOLOGY

H1N1 viruses and H3N2 viruses are primary sub-types of Influenza A that have circulating around the world since 1977. Occasionally, H1N2 viruses, which are theorized to be a genetic reassortment between H3N2 and H1N1 viruses also has been detected during some influenza seasons. Influenza B viruses are also in circulation globally.[2]

The influenza season is typically during the fall or winter months. However, the peak of the season can occur as late as April or May. Rates of influenza infection are typically highest among children; however, the rates of influenza-related complications are usually higher among persons older than 65 years and persons with comorbidities.[2]

Influenza is usually spread from an infected person to others via coughing or sneezing. The infected person may be able to start infecting others one day prior to symptoms and up to five days after showing symptoms. People can also become infected by touching infected items then touching their nose or mouth.[3]

Influenza infections frequently increase the hospitalization rates among individuals older than 65 years. One retrospective study of managed-care data from 1996-2000 found that there were approximately 560 influenza-associated hospitalizations per 100,000 persons in contrast to approximately 190 hospitalizations per 100,000 healthy elderly persons. For individuals aged 50 to 64 years with underlying medical conditions, hospitalization also increased. significantly.[2]

Influenza also contributes increased mortality in individuals with pneumonia or AIDS. Among individuals older than 65 years, there was an estimated yearly average of 32,651 influenza-related deaths from 1976 through 2001. And, for individuals with AIDS, the risk for influenza-related death was estimated at 94 to 146 deaths per 100,000 persons in contrast to 0.9 to 1.0 deaths per 100,000 persons aged 25 through 54 years and 64 to 70 deaths per 100,000 persons for individuals older than 65 years in the general population.[2]

During the previous influenza pandemic of 1918 – 1919 and 1957 – 1958, there were increased influenza-associated deaths among pregnant women; and, some case studies indicated that pregnancy may increase the risk of influenza complications in the mother. Several studies have indicated increased number of medical visits by pregnant women for respiratory illness during influenza seasons. In one study, 0.4% of the pregnant women were hospitalized and 25% visited a healthcare provider during pregnancy for a respiratory illness. Also, it was noted that the rate of third-trimester hospital admissions during the influenza season was five times higher than the rate during the influenza season in the year prior pregnancy and more than twice as high as the rate during the non-influenza season. The study further noted an extra 1,210 hospital admissions in the third trimester per 100,000 pregnant women who had comorbidities and 68 admissions per 100,000 women without comorbidities. Other studies examining the infants of these pregnant women did not note higher rates of low birth weight, congenital abnormalities, or low Apgar scores compared with infants born to uninfected women.[2]

However, infants and young children are higher risk for influenza-associated hospitalization than older children. Infants and young children have similar rates to high-risk groups for influenza-related complications. The estimated rate of influenza-associated hospitalization for children under five years from 1979 through 2001 in the United States was 108 hospitalizations per 100,000 person-years. For infants less than six months, the annual hospitalization rate for influenza is 240-720 per 100,000 children. This rate decreases to 20 per 100,000 children in those aged two through five years. And, for children less than five years, who have high-risk medical conditions, the hospitalization rate for influenza is 250-500 per 100,000 children.[2]

Typically, these hospitalizations do not result in death. The estimated annual average for children under five during the 1990s was 92 influenza-related deaths (0.4 deaths per 100,000 persons) in contrast to 32,651 deaths (98.3 per 100,000 persons) among adults older than 65 years. The annual pediatric mortality from influenza in the United States has ranged around 84 for the 2004-2005 and 2007- 2008 influenza seasons.[2]

Globally, these influenza outbreaks result in about three to five million cases of severe illness, and approximately 250,000 to 500,000 deaths annually. Unlike in more temperate areas, the influenza viruses circulate throughout the year with one or two peaks during the rainy seasons in tropical countries.[4]

## SYMPTOMS/COMPLICATIONS

The typical incubation period for influenza is one to four days with an average of two days. Adults can shed virus from one day prior to symptoms through five to 10 days after symptom onset. These symptoms usually resolve after three to seven days; although, the cough may persist for longer than two weeks. In contrast, children can become infectious several days prior to symptoms and continue to shed virus for more than 10 days after illness onset. And, the severely immunocompromised can continue to shed influenza virus for weeks to months after infection.[2]

The usual symptoms of influenza include the abrupt onset of high fever, muscle pain, headache, malaise, a nonproductive couth, rhinitis, and sore throat. Children may also develop otitis media and nausea and vomiting.

Influenza infections can also lead to complications including primary influenza viral pneumonia, secondary bacterial pneumonia, sinusitis, otitis media, exacerbation or pulmonary or cardiac disease, and contribute to viral or bacterial coinfections. Influenza infections in young children can also mimic bacterial sepsis and produce febrile seizures. Also, deaths among children coinfected with influenza and *Staphylococcus aureus* (especially MRSA) have also been on the increase over the past 4 influenza seasons. Occasionally, influenza infections have also caused encephalopathy, pericarditis, transverse myelitis, myocarditis, myositis, and Reye syndrome.[2]

## PREVENTION

Annual vaccination is the most effective prevention strategy for influenza. These yearly vaccinations should begin in September and continue through January and beyond because the peak influenza activity can occur in January and later months.[2]

There are antiviral drugs that can be used for chemoprophylaxis or treatment; however, viral resistance can quickly develop. Frequent handwashing and proper respiratory hygiene are also helpful reducing the spread of respiratory diseases; however, there have been insufficient studies to determine if these measures can reduce the transmission of influenza.[2]

Influenza vaccine can be administered to all individuals (except for those with contraindications) older than six months. These contraindications can include severe allergy to chicken eggs, prior severe reaction to an influenza vaccination, previous development of Guillian-Barré syndrome (GBS) within six weeks of getting an influenza immunization, infants less than 6 months, and individuals with a current moderate or severe illness with a fever who should wait to get vaccinated until their symptoms decrease. [3] There are two types of influenza vaccines: the trivalent inactivated influenza vaccine (TIV) and the live, attenuated influenza vaccine (LAIV) (FluMist®).

The TIV can be used for individual older than six months including those with high-risk conditions. The TIV may produce soreness, redness, and swelling at the injection site. It may also produce a low-grade fever and aches for one to two days.

The LAIV can be used for healthy, non-pregnant individuals aged two through 49 years. However, the LAIV is not recommended for persons with underlying medical conditions or for persons working with persons with immunocompromised immune systems.[2] LAIV can produce a runny nose, wheezing, headache, vomiting, muscle aches, and fever in children. In adults, the LAIV may produce a runny nose, headache, sore throat, and cough.[3]

Each vaccine contains three influenza viruses including one A (H3N2) virus, one A (H1N1) virus, and one B virus. The virus strains included in the 2008-2009 trivalent vaccine are A/Brisbane/59/2007 (H1N1)-like, A/Brisbane/10/2007 (H3N2)-like, and B/Florida/4/2006-like antigens. The viruses in the vaccines are changed each year based on international surveillance and estimations about which types and strains of viruses will be circulating that year.[2]

The effectiveness of the vaccine depends on the age and health status of the person as well as the similarity of the virus strains in the vaccine to the virus in circulation. Approximately two weeks after vaccination, antibodies will have developed against the influenza virus in the vaccine.[3]

## TREATMENT

There are four prescription medications in the United States available for treatment and chemoprophylaxis of influenza. These medications include oseltamivir, zanamivir, amantadine and rimantadine.[2]

However, since January 2006, only the neuraminidase inhibitors, oseltamivir (Tamiflu®) and zanamivir (Relenza®) have been recommended for influenza because of the widespread resistance of Influenza A (H3N2) to the adamantanes, amantadine and rimantadine.[2]

The neuraminidase inhibitors have activity against both Influenza A and B viruses. In contrast, the adamantanes have activity only against influenza A virus.

During the 2007-2008 the period of rampant influenza, a significant increase in oseltamivir resistance was noted among influenza A (H1N1) viruses around the world. In fact, 10.9% of H1N1 viruses tested in the U.S. were resistant to oseltamivir during 2007-2008. However, oseltamivir can still be used for treatment of H3N2 viruses and Influenza B.[2,5]

In other cases with H1N1 influenza or an unidentified influenza virus, zanamivir may be preferred. For individuals who can not take zanamivir (children under 7 years, persons with chronic underlying airway disease, and those unable to use a zanamivir inhalation device) or zanamivir is unavailable, combination treatment with oseltamivir and rimantadine is an acceptable alternative. Amantadine can also be substituted for rimantadine if rimantadine is unavailable.[2,5]

As of December 2008, of the 50 H1N1 viruses from 12 states that had been tested in the United States, 98% were resistant to oseltamivir but still susceptible to zanamivir, amantadine and rimantadine. These particular viruses did not appear to cause any different or more severe illness that the oseltamivir-sensitive H1N1 viruses. On a positive note, the Influenza A (H3N2) viruses and B viruses remain susceptible to oseltamivir; and, the oseltamivir-resistant H1N1 viruses are antigenically similar to the H1N1 virus strain represented in 2008-09 influenza vaccine.[5]

## INFECTION CONTROL

Important infection control measures for influenza include:

1. Annual influenza vsaccinations for all eligible patients and healthcare workers,
2. Use of Standard and Droplet Precautions for infected individuals,
3. Active surveillance and influenza testing of new illness cases,
4. Restriction of ill visitors and healthcare workers,
5. Rapid administration of antiviral medications for treatment and chemoprophylaxis during outbreaks, and
6. Respiratory hygiene and cough etiquette.

For influenza vaccinations, either TIV or LAIV may be used for healthcare workers. However, LAIV is preferred for use with healthcare workers who work with severely immunocompromised patients. If healthcare workers do receive LAIV, they should not work around severely immunocompromised patients for seven days after the vaccination.[6]

Standard and Droplet Precautions should be instituted to reduce the potential spread of influenza from infected individuals. Because influenza is primarily transmitted person to person via large droplets from coughs and sneezes, close contact is usually considered within 6 feet of the infected person. Gloves and surgical masks should be worn during the care of patients with suspected or confirmed influenza for five days after the onset of illness. The patient should wear a surgical mask during transport outside of their room.[6]

Active surveillance for respiratory illness and rapid influenza testing are also essential to identify outbreaks early and to prevent the spread of influenza. Depending on the situation, rapid diagnostic tests, immunofluorescence, and viral cultures can be done to detect and confirm influenza infections.

Visitors with respiratory symptoms should be discouraged from visiting patients until at least 10 days after the onset of symptoms. Healthcare workers with respiratory symptoms should be removed from direct patient contact for five days after symptom onset.[6]

Antivirals, such as oseltamivir (Tamiflu®) and zanamivir (Relenza®), can also be important in preventing and treating cases of influenza. If possible, it is better to do rapid influenza testing prior to the administration of the antivirals to ensure that the medication will be effective.

Respiratory hygiene and proper cough etiquette can also be useful in controlling an influenza outbreak. Posting signs of influenza symptoms and what to do can be helpful. The use of tissues and masks can help reduce the spread of influenza. Hand washing with either soap and water or alcohol-based hand rubs is also very important in reducing the spread of influenza.

### Surveillance

Influenza surveillance in the United States is carried out by the Epidemiology and Prevention Branch in the Influenza Division at the CDC. The Epidemiology and Prevention Branch collects, compiles and analyzes information on influenza activity throughout the year and produces a weekly report from October through early May. This system is a collaborative effort between the CDC and state and local health departments, public health and clinical laboratories, vital statistics offices, healthcare providers, clinics and emergency departments. This surveillance system collects information in five categories from nine different data sources which allows the CDC to determine when and where influenza activity is occurring, follow influenza- related illness, track which influenza viruses are circulating, determine changes in influenza viruses, and determine the impact influenza is having on U.S. mortality.[7]

The first category of influenza surveillance is viral surveillance. Approximately 80 U.S. World Health Organization Collaborating Laboratories and 70 National Respiratory and Enteric Virus Surveillance System (NREVSS), scattered throughout the United States are involved in virologic surveillance for influenza. These laboratories are also involved in surveillance for novel Influenza A viruses.

The second category of influenza surveillance is outpatient illness surveillance. With this surveillance, information from patient visits to health care providers for influenza-like illness is collected through the US Outpatient Influenza-like Illness Surveillance Network (ILINet). The ILINet consists of approximately 2,400 healthcare providers in 50 states reporting approximately 16 million patient visits each year.

The third category of influenza surveillance is mortality surveillance which is done through two systems. The first system is the 122 Cities Mortality Reporting System in which the vital statistics offices in 122 cities report the total number of death certificates received and the number of those deaths due to pneumonia or influenza. The percentage of these deaths due to pneumonia or influenza is compared to a seasonal baseline and an epidemic threshold value which is calculated each week. The second system is for surveillance of influenza-associated pediatric mortality. An influenza-associated death in people under 18 was added as a nationally notifiable condition in 2004. The laboratory-confirmed influenza-associated deaths in children are reported through the Nationally Notifiable Disease Surveillance System.

The fourth category of influenza surveillance is hospitalization surveillance which is monitored by two systems. The first system is the Emerging Infections Program (EIP) which conducts surveillance for laboratory-confirmed influenza related hospitalizations in children (less than 18 years) and adults in 60 counties covering 12 metropolitan areas of 10 States (San Francisco CA, Denver CO, New Haven CT, Atlanta GA, Baltimore MD, Minneapolis/St. Paul MN, Albuquerque NM, Las Cruces, NM, Albany NY, Rochester NY, Portland OR, and Nashville TN). The second system is the New Vaccine Surveillance Network (NVSN) which provides population- based estimates of laboratory-confirmed influenza hospitalization rates for children under five residing in three counties: Hamilton County OH, Davidson County TN, and Monroe County NY.

The fifth category of influenza surveillance is the summary of the geographic spread of influenza in which state health departments report the estimated level of spread of influenza activity in their states each week through the state and territorial epidemiologists reports.[7]

At the global level, the World Health Organization also has the WHO Global Influenza Programme. The mission of the Programme is to reduce death and disease due to annual influenza epidemics and to prepare for the next influenza pandemic. The Programme focuses on four major areas which include (1) global influenza surveillance for accurate and timely recommendations on influenza vaccine composition, (2) enhancement of global and national pandemic preparedness which includes initial outbreak investigation and coordination of rapid response, (3) preparation and publication of technical and standard setting documents on influenza surveillance and control, and (4) providing international leadership in the coordination of implementation and advocacy of the Global Agenda on Influenza Surveillance, Prevention and Control.[8]

The World Health Organization also has the WHO Global Influenza Surveillance Network. The main components of the Network are National Influenza Centres (NICs) which take samples from patients with influenza-like- illness and submit representative isolates to the four WHO Collaborating Centres (WHO CCs) (in Australia, Japan, United Kingdom, and United States) for antigenic and genetic analysis. NICs, WHO CCs and WHO compose the WHO Global Influenza Surveillance Network. There are currently 125 institutions in 96 countries which serve as NICs; and, these NICs collect more than 175,000 patient samples and submit approximately 2,000 viruses to the WHO CCs for analysis. The Network allows the WHO to recommend twice a year the content of the influenza vaccine for the subsequent influenza season. More than 250 million doses of influenza vaccine are produced annually with this updated content. The Network also serves as a global alert mechanism for the emergence of influenza viruses with pandemic potential. The Network has also contributed greatly to the understanding of influenza epidemiology.[9]

Finally, the WHO also had the Communicable Disease Global Atlas which allows for analysis and comparison of standardized data and statistics for infectious diseases at the country, regional, and global levels.[10]

## CONCLUSION

Seasonal influenza seldom attracts the media attention that the current Swine Flu epidemic has had in the past months or inspires the level of concern that the 1918 Influenza pandemic has over the years. But, seasonal influenza greatly contributes to the overall morbidity and mortality around the world yearly in contrast to the infrequent pandemics. In most instances, seasonal influenza can be readily controlled with proper vaccination, chemoprophylaxis and treatment, and infection control measures. The global surveillance systems also play a major in detecting and controlling seasonal influenza; and, in controlling seasonal influenza, these surveillance systems have a major role in detecting the next possible influenza pandemic.
